# The intact postsynaptic protein neurogranin is reduced in brain tissue from patients with familial and sporadic Alzheimer’s disease

**DOI:** 10.1007/s00401-018-1910-3

**Published:** 2018-09-22

**Authors:** Hlin Kvartsberg, Tammaryn Lashley, Christina E. Murray, Gunnar Brinkmalm, Nicholas C. Cullen, Kina Höglund, Henrik Zetterberg, Kaj Blennow, Erik Portelius

**Affiliations:** 1 Department of Psychiatry and Neurochemistry, Institute of Neuroscience and Physiology, The Sahlgrenska Academy at the University of Gothenburg, Sahlgrenska University Hospital/Mölndal, S-431 80 Mölndal, Sweden; 2 Queen Square Brain Bank for Neurological Disorders, Department of Movement Disorders, UCL Institute of Neurology, London, UK; 3 Department of Neurodegenerative Disease, UCL Institute of Neurology, Queen Square, London, UK; 4 UK Dementia Research Institute at UCL, London, UK; 5 Clinical Neurochemistry Laboratory, Sahlgrenska University Hospital, Mölndal, Sweden; 6 Department of Neurobiology, Care Sciences and Society, Center for Alzheimer Disease Research, Neurogeriatrics Division, Karolinska Institutet, Novum, Huddinge, Stockholm, Sweden

**Keywords:** Neurogranin, Alzheimer’s disease, Brain tissue, Familial Alzheimer’s disease, Mass spectrometry

## Abstract

Synaptic degeneration and neuronal loss are early events in Alzheimer’s disease (AD), occurring long before symptom onset, thus making synaptic biomarkers relevant for enabling early diagnosis. The postsynaptic protein neurogranin (Ng) is a cerebrospinal fluid (CSF) biomarker for AD, also in the prodromal phase. Here we tested the hypothesis that during AD neurodegeneration, processing of full-length Ng into endogenous peptides in the brain is increased. We characterized Ng in post-mortem brain tissue and investigated the levels of endogenous Ng peptides in relation to full-length protein in brain tissue of patients with sporadic (sAD) and familial Alzheimer’s disease (fAD), healthy controls and individuals who were cognitively unaffected but amyloid-positive (CU-AP) in two different brain regions. Brain tissue from parietal cortex [sAD (*n* = 10) and age-matched controls (*n* = 10)] and temporal cortex [sAD (*n* = 9), fAD (*n* = 10), CU-AP (*n* = 13) and controls (*n* = 9)] were included and all the samples were analyzed by three different methods. Using high-resolution mass spectrometry, 39 endogenous Ng peptides were identified while full-length Ng was found to be modified including disulfide bridges or glutathione. In sAD parietal cortex, the ratio of peptide-to-total full-length Ng was significantly increased for eight endogenous Ng peptides compared to controls. In the temporal cortex, several of the peptide-to-total full-length Ng ratios were increased in both sAD and fAD cases compared to controls and CU-AP. This finding was confirmed by western blot, which mainly detects full-length Ng, and enzyme-linked immunosorbent assay, most likely detecting a mix of peptides and full-length Ng. In addition, Ng was significantly associated with the degree of amyloid and tau pathology. These results suggest that processing of Ng into peptides is increased in AD brain tissue, which may reflect the ongoing synaptic degeneration, and which is also mirrored as increased levels of Ng peptides in CSF.

**Electronic supplementary material:**

The online version of this article (10.1007/s00401-018-1910-3) contains supplementary material, which is available to authorized users.

## Introduction

Alzheimer’s disease (AD) is the most common form of dementia, affecting tens of millions of people worldwide and with numbers increasing each year [1]. AD is a neurodegenerative disorder characterized by certain neuropathological hallmarks in the brain including extracellular plaques consisting of amyloid β (Aβ) peptides and intracellular neurofibrillary tangles composed of hyperphosphorylated tau (p-tau) protein [9]. Most AD patients have no known cause and are termed sporadic (sAD), but around 1% are due to autosomal dominant mutations in genes related to amyloid metabolism, e.g., *APP*, *PSEN1* and *PSEN2*, causing the hereditary form called familial AD (fAD) [6, 13, 55]. In contrast to sporadic AD, which usually presents after the age of 65, the onset of fAD is generally much earlier.

Even at early stages of the disease, dysfunction and loss of synapses are directly linked to cognitive symptoms such as memory disturbances and are thought to occur earlier than neuronal loss [18]. In fact, the degree of dementia has been found to be more associated with synaptic loss compared to amyloid plaques and tangles [8, 16, 37, 52, 58]. In addition, studies using immunohistochemistry and immunoelectronmicroscopy also suggest that synaptic loss occur without clear relation to plaque pathology [8, 36]. Synaptic loss is especially pronounced in certain areas of the brain such as the hippocampus [52, 53]. Consequently, synaptic proteins have the potential to be highly suited as biomarkers for early AD diagnosis in addition to monitoring disease progression and evaluating novel disease-modifying therapeutics.

Neurogranin (Ng) is a 78-amino acid-long postsynaptic protein that plays a critical role in long-term potentiation (LTP). LTP is thought to be crucial for the formation of long-term memories, through regulating the concentration of calmodulin by responding to intracellular calcium levels following neuronal excitation [4, 20, 25, 63]. Ng is able to bind to calmodulin via an IQ motif (amino acid 33–46) which is well conserved among other calmodulin-binding proteins, such as the pre-synaptic protein growth-associated protein 43 (GAP-43). In the brain, Ng expression is localized to dendritic spines of neurons in the amygdala, hippocampus, cerebral cortex, and other associative cortical areas [10, 20]. In mice, both Ng mRNA and protein concentrations in the hippocampus decrease with age and are related to central nervous system (CNS) dysfunction [41]. In addition, knockdown models display reduced LTP as well as impaired cognition [23] while upregulation improves LTP along with cognitive performance [65].

It is well established that cerebrospinal fluid (CSF) Ng is increased in sAD compared to healthy controls [17, 24, 28, 30, 32, 33, 45, 46]. Increased CSF Ng concentrations can also be used to distinguish patients with mild cognitive impairment (MCI) that will convert to sAD from those that remain stable [30, 32, 38, 46]. However, data from brain tissue indicate a decrease of Ng concentrations in both the frontal cortex [15, 49], parietal cortex [49] and hippocampus [15]. We have previously shown that Ng in brain tissue is present both as intact full-length protein and as a variety of endogenous C-terminal peptides [32]. Therefore, we aimed to characterize Ng in brain tissue and to quantify full-length Ng and Ng peptides in post-mortem brain tissue of patients with sAD, fAD, healthy controls as well as cognitively unaffected amyloid-positive (CU-AP) individuals, which have neuropathological changes beyond normal levels for their age. We also tested the hypothesis that during the neurodegenerative process of AD, higher amounts of endogenous Ng peptides are generated in the brain through processing of full-length Ng compared to brains without AD neuropathology. Here, we present results of Ng in post-mortem brain tissue from two different cohorts measured by three independent methods, showing that full-length Ng in the brain has post-translational modifications (PTM) and that there is a shift from full-length Ng to peptides in both sAD and fAD compared to CU-AP and controls.

## Materials and methods

### Individuals included in study 1

Post-mortem brain tissue samples from the superior parietal gyrus of individuals with sAD (*n* = 10) and healthy controls (*n* = 10), obtained from the Netherlands Brain Bank, were stored at − 80 °C pending biochemical analysis. All sAD patients fulfilled Braak stages V or VI and the controls fulfilled Braak stages 0 or I in accordance with the Braak and Braak criteria [11]. As this material was collected and scored before 2012, the 2012 National Institute on Ageing (NIA) and the Alzheimer’s Association (AA) guidelines for neuropathologic assessment of AD [26] were not used. Controls were assessed by retrospective telephone interviews with the next of kin, to assure that they had no cognitive symptoms. Demographics of the patients included in study 1 are shown in Table 1 and full demographics are detailed in Online Resource 1.

**Table 1:** Demographics and clinical characteristics of subjects included in study 1 and 2

	sAD	Control	fAD	CU-AP
**Study 1**	*n* = 10	*n* = 10		
Gender, *n*, female/male (% female)	6/4 (60)	6/4 (60)		
Age at death	77 [72–79]	72.5 [69.5–80.5]		
Post-mortem delay, (h)	5 [4–6]	7 [6–7]		
Braak stage 0–I/II–IV/V–VI	0/0/10	10/0/0		
**Study 2**	*n* = 9	*n* = 9	*n* = 10	*n* = 13
Gender, *n*, female/male (% female)	3/6 (33)	5/4 (56)	6/4 (60)	9/4 (69)
Age at onset	60 [52–69]^§^	n/a	43 [36–53]	n/a
Duration, (years)	11.5 [10–15]	n/a	9 [5–13]	n/a
Age at death	72 [66–80]*	82 [70–85]^¤^	54 [46–65]^#^	88 [84–91]
Post-mortem delay, (h)	65 [47–92]	75 [40–86]	38 [26–65]^##^	78 [39–102]
Brain weight, (g)	1116 [1009–1244]^†^	1330 [1250–1474]	1108 [880–1320]^¤¤^	1264 [1192–1421]
Braak stage 0–I/II–IV/V–VI	0/0/9	7/2/0	0/0/10	1/12/0
Thal stage 0–1/2–4/5–6	0/0/9	8/1/0	0/0/10	1/9/3
CERAD score 0/A/B/C	0/0/0/9	6/2/0/1	0/0/0/10	2/6/5
ABC score, minimum–maximum	A3B3C3	A0B0C0–A2B1C1/A1B2C1	A3B3C3	A1B2C2–A3B2C2
Mutation, *PSN1*/*APP*			8/2	
ELISA, (ng/mg) total protein	253.9 [137-346.2]	398.1 [305.9–631.8]	291.2 [169.9–363.3]^###^	518.7 [440.1–741.7]

Differences between groups in study 1 were assessed using Mann–Whitney *U* test. Comparisons between groups in study 2 were performed using Kruskal–Wallis test with data adjusted for post-mortem delay, followed by pairwise Mann–Whitney *U* tests if significant. The data are presented as median and [interquartile range]

^§^sAD vs fAD *p* = 0.003; *sAD vs CU-AP *p* = 0.033; ^†^sAD vs controls *p* = 0.013; ^¤^fAD vs control *p* = 0.032; ^¤¤^fAD vs control *p* = 0.025; ^#^fAD vs CU-AP *p* < 0.0001; ^##^fAD vs CU-AP *p *= 0.029; ^###^fAD vs CU-AP *p* = 0.017

### Individuals included in study 2

Post-mortem brain samples from temporal cortex of patients with sAD (*n* = 9), fAD (*n* = 10), CU-AP (*n* = 13) and healthy controls (*n* = 9) were obtained from the Queen Square Brain Bank, UCL Institute of Neurology, London, UK. AD patients fulfilled the clinical NINCDS criteria for probable AD [39] and met the 2012 NIA-AA guidelines for neuropathologic assessment of AD [26]. Thal phases were determined as a measure of the spread Aβ pathology throughout the brain as described [60]. Braak stages were scored according to the Braak and Braak criteria [11]. Braak tau neurofibrillary tangle staging (PHF-1) and the Consortium to Establish a Registry for AD (CERAD) neuritic plaque score (thioflavin stain) were used to classify AD neuropathology into four groups as described previously (Hyman et al. 2012): no (or negligible) AD neuropathology (0), low level AD (A), intermediate-level AD (B), and high-level AD (C). In addition, an ABC score that incorporates histopathologic assessments of Aβ deposits (A), staging of neurofibrillary tangles (B) and scoring of neuritic plaques (C) was calculated as described previously [42]. fAD patients had mutations in either *PSEN1* (*n* = 8) or *APP* (*n* = 2) genes. The *PSEN1* mutations included R278I, E120K, A434T + T291A, I202F, E184D, S132A, and intron 4 and all *APP* mutations were V717I. Due to the numbers of different mutations in the fAD group the cases were grouped together for analysis. The cognitively normal status of the control cases used in this study was confirmed from assessment forms received at brain donation. The assessment forms are completed by both the person donating their brain and/or by relatives or next of kin. We also have access to all the medical records that were summarized by a neurologist. The CU-AP patients were cases who had died from acute cardiac or malignant disease, without history of dementia, or psychiatric or neurological diseases where the autopsy examination revealed AD-like pathology beyond what can be considered normal for age. A summary of case demographics of the cases included in study 2 is shown in Table 1, with full demographics detailed in Online Resource 1.

Both studies were conducted according to the provisions of the Helsinki Declaration. All subjects gave written informed consent for the use of their clinical data for research purposes, and the local Ethical Committees at the respective university approved each study.

### Generation of anti-Ng antibodies

The monoclonal anti-Ng antibodies Ng2 and Ng3 have been described previously [32], while Ng36 was generated using the same protocol, but with KLH-conjugated peptide Ng63–75 (Caslo ApS Denmark) as immunogen. All of the antibodies were purified using a protein G column (GE Healthcare).

### Homogenization of brain tissue for western blot, mass spectrometric analysis and immunoassay

Brain tissue (100 ± 10 mg) (superior parietal gyrus or temporal cortex) were homogenized on ice in Tris-buffered saline (TBS) (1:5 weight:volume ratio) containing complete protease inhibitor (Roche Diagnostics GmbH, Mannheim, Germany). The homogenized tissue was further diluted in TBS (1:5) followed by centrifugation at 31,000*g* at + 4 °C for 1 h. The supernatant (TBS fraction) was removed and stored at − 80 °C pending analysis. Total protein concentration of all homogenized samples was determined using the DC™ Protein Assay (Bio-Rad Laboratories) according to the manufacturer’s instructions. The samples were diluted in PBS (1:40) prior to immunoprecipitation.

### Hybrid immunoaffinity–mass spectrometry

4 µg of the anti-Ng antibodies Ng2 and Ng3 were separately added to 25 μL M-280 Dynabeads (Sheep anti-mouse IgG, Invitrogen) according to the manufacturer’s product description and cross-linked as previously described [12]. Ng2- and Ng3-coated beads were used for immunoprecipitation of brain extracts to which Tween 20 (final concentration 0.025%) was added and incubated. Beads and samples were transferred to a KingFisher magnetic particle processor (polypropylene tubes, Thermo Scientific, Waltham, MA, USA) for automatic washing and elution of full-length and Ng peptides. Eluted Ng was collected and dried in a vacuum centrifuge and re-dissolved in 5 μL 0.1% formic acid (FA) in 20% acetonitrile (ACN) and subsequently analyzed using a Bruker Daltonics UltraFleXtreme matrix assisted laser desorption/ionization time-of-flight/time-of-flight (MALDI TOF/TOF) mass spectrometer (Bruker Daltonics, Bremen, Germany) or high-resolution tandem mass spectrometry (MS/MS) using a Dionex Ultimate 3000 nanoflow liquid chromatography (LC) system (Thermo Fisher Scientific). A detailed description about LC–MS/MS and database searches that were performed can be found in Online Resource 2. All solvents used were of HPLC grade. In both studies in which MALDI TOF/TOF MS was used, the custom Ng peptide RKKIKSGERGRKGPGPGGPGGAGVARGGAGGGP (corresponding to Ng43–75), with all glycines fully labeled with ^13^C (theoretical molecular mass: 3011 Da; CASLO, Lyngby, Denmark), was added to the tissue homogenate during sample preparation and was used as an internal standard. Samples were analyzed blinded (that is, without knowledge of clinical diagnosis). All calculated concentrations were normalized to total protein concentration of the sample.

### Reduction and alkylation of full-length neurogranin

Full-length Ng from brain tissue was first purified according to the hybrid immunoaffinity–mass spectrometry (HI–MS) procedure. After vacuum centrifugation, the samples were dissolved in 5 μL 20% ACN and vortexed for 1 h. Thereafter 15 µL 50 mM NH_4_HCO_3_ was added and samples were vortexed. Then 20 μL 10 mM dithiothreitol (DTT) in 50 mM NH_4_HCO_3_ was added to the samples followed by a 3-min incubation at + 90 °C. The samples were allowed to cool to room temperature for 30 min after which 40 μL 10 mM iodoacetamide in 50 mM NH_4_HCO_3_ was added. Samples were then incubated at room temperature in the dark for 30 min after which they were dried in a vacuum centrifuge and stored at − 80 °C pending MS analysis. Negative control samples were only incubated 3 min at 90 °C without the addition of DTT prior to drying in a vacuum centrifuge.

### Western blot

1 µg of total protein was mixed with XT sample buffer (Bio-Rad Laboratories) and XT reducing agent (Bio-Rad Laboratories) before boiling 5 min at 95 °C. Samples and full-length recombinant calibrator Ng–Myc–DKK fusion protein (Origene, product no TP301209) ranging from 1.56 to 25 ng were electrophoresed on 12% Criterion XT Bis–Tris Gel using the Criterion cell tank (Bio-Rad Laboratories). The proteins were transferred to 0.2 µm nitrocellulose membrane (Amersham), using the semi-dry blotting technique. Blocking was performed for 1 h at room temperature using 5% blotting-grade blocker (Bio-Rad Laboratories) in phosphate-buffered saline (0.01 M phosphate buffer, 0.14 M NaCl, pH 7.4; PBS) containing Tween 20 (Bio-Rad Laboratories, final concentration 0.05%; PBS-Tween). Incubations with the monoclonal Ng36 diluted 1:1250 in 1% blotting-grade blocker in PBS-Tween or no primary antibody (negative control) was performed overnight at + 4 °C. The membranes were washed for 3 × 10 min in PBS-Tween and then incubated for 1 h at room temperature with goat anti-mouse IgG (H + L) poly-HRP secondary antibody HRP (0.5 mg/mL) (Thermo Fisher Scientific) diluted 1:15,000 in PBS-Tween containing 1% bovine serum albumin (BSA). Following 3 × 10 min washes in PBS-Tween, the membranes were developed for 2 min with ECL Select™ Western Blotting Detection Reagent (GE Healthcare) according to the manufacturer’s instructions. The emitted signal was detected by a Fujifilm LAS-3000 System (FUJIFILM Corporation) and protein bands were quantified using ImageJ software version 1.51j8 (Rasband, WS, ImageJ; National Institutes of Health, Bethesda, MD, http://rsb.info.nih.gov/ij). SOFTmax^®^ Pro 4.0 (Molecular Devices Corporation, Sunnyvale, CA, USA) and a fitted four-parameter logistic model calibration curve were used to quantify Ng concentration of samples. Samples that were below the lowest point of the standard curve but still visible were given a value of half the lowest concentration, i.e., 0.78 ng. A standard curve, negative controls, quality control sample of brain tissue homogenate and molecular size marker SeeBlue Plus2 Pre-stained protein standard (Thermo Fisher Scientific) was included on all gels.

### Sandwich ELISA method of Ng

Ng36 was used as a capturing antibody and was coated on Nunc maxisorp 96-well microtiter plates at a final concentration of 0.5 μg/mL (100 µL/well) in NH_4_HCO_3_ buffer, pH 9.6, overnight at + 4 °C. After washing with PBS-Tween 4**×**350 µL, the remaining protein binding sites were blocked with 1% BSA in PBS-Tween for 1 h at room temperature (250 μL/well). Thereafter, plates were washed with PBS-Tween 4**×**350 µL. Full-length GST-tagged recombinant Ng calibrators with concentrations ranging between 7 and 940 pg/mL, blanks and TBS brain samples (diluted 1:10,000, 100 μL/well) were incubated in duplicate for 3 h at room temperature, 350 rpm, followed by washing with 4 × 350 µL PBS-Tween. Detector antibody, biotinylated Ng2 final concentration 0.5 μg/mL (100 µL/well) in 1% BSA PBS-Tween, was added followed by incubation for 1 h at room temperature and washing with 4**×**350 µL PBS-Tween. Enhanced streptavidin-HRP (KemEnTech) was diluted according to the manufacturer’s instructions and added (100 µL/well) and incubated 30 min at room temperature. After washing with 4**×**350 µL PBS-Tween, TMB substrate (BioRad Laboratories, 100 µL/well) was added to produce the color reaction. After 20 min in darkness, the reaction was stopped by addition of 100 μL of 0.2 M H_2_SO_4_, and following 1 min incubation at room temperature, 450 rpm, the absorbance was measured at 450 nm (reference wavelength 650 nm) using an ELISA plate reader (Sunrise, Tecan Trading AG, Switzerland). A fitted four-parameter logistic model was used as the calibration curve and the blank was included as zero concentration of Ng (SoftMax Pro v. 4.0).

### Statistical analysis

In the first study cohort containing individuals with sAD and controls, we analyzed differences in total full-length Ng, meaning the sum of all Ng1–78 with various modifications and the peptide-to-total full-length Ng ratio of all Ng peptides identified by HI–MS. The data in the second study cohort, containing individuals with sAD, fAD, CU-AP, and controls, were analyzed in the same way as for the first study. Because there were more than two groups in this cohort, we first performed the non-parametric Kruskal–Wallis test for differences between groups, followed by post hoc pairwise Mann–Whitney *U* tests for individual group differences if the Kruskal–Wallis test was significant. All Ng measures in this study were first adjusted for post-mortem delay and age. Additionally, we analyzed the association between Ng and Aβ and tau pathology in the second cohort by calculating the Spearman correlation between HI–MS/WB/ELISA values and both Braak and Thal staging. We tested for group differences in Ng values between Braak stages 0–I, II–IV, V–VI, and separately between Thal scores 0–1, 2–3, 4–5, and again separately between CERAD scores 0, A, B, C—each using the Kruskal–Wallis test with post hoc pairwise Mann–Whitney *U* tests if the main effect was significance. All tests were two-sided with a significance level set to *p *< 0.05. *p* values for each family of comparisons were adjusted for multiple comparisons using Holm’s method to control the family-wise error rate. Statistical analysis was performed using GraphPad Prism 7 (GraphPad Software, La Jolla, USA) and the R programming language (v. 3.4.3).

## Results

### Neurogranin is present as both endogenous peptides and modified full-length form

**Fig. 1:**
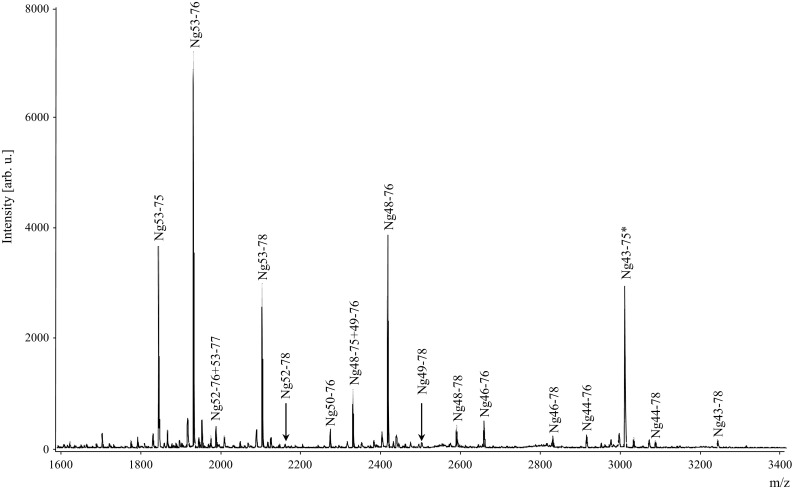
Hybrid immunoaffinity–mass spectrometric characterization of endogenous neurogranin peptides in human brain tissue. Several short endogenous C-terminal peptides were repeatedly detected in human parietal cortex using the monoclonal antibodies Ng2 + Ng3 and MALDI TOF/TOF. Ng43–75* represents internal standard fully labeled with ^13^C

**Fig. 2:**
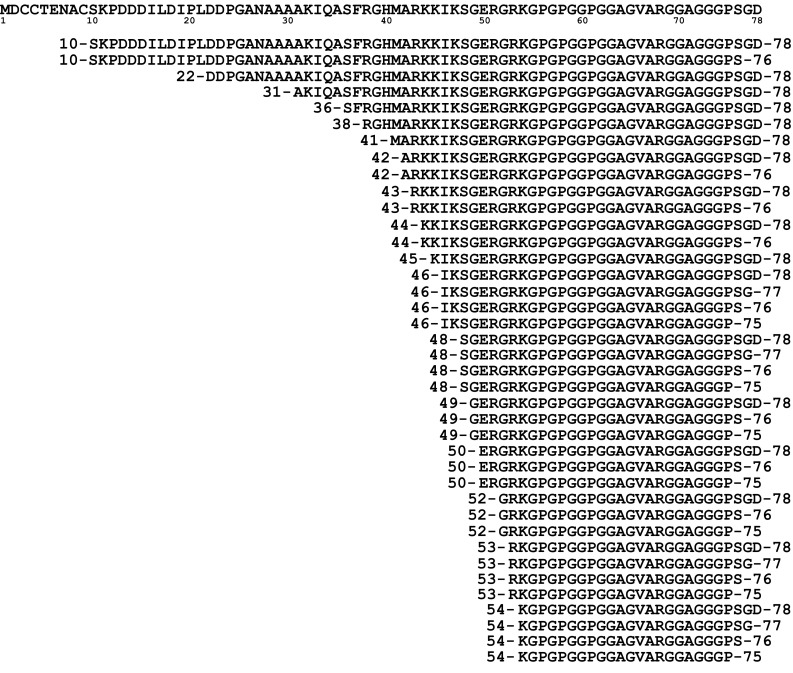
Summary of all identified neurogranin peptides in human brain tissue. In total, 39 endogenous Ng peptides were identified using a combination of HI–MS and high-resolution mass spectrometry

**Fig. 3:**
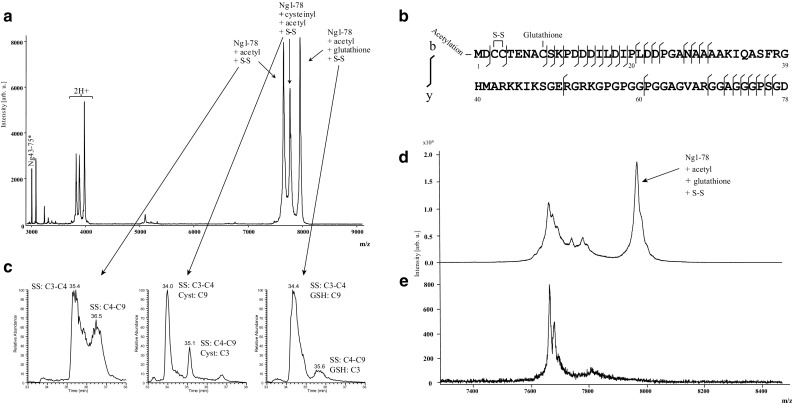
Hybrid immunoaffinity–mass spectrometric characterization of full-length neurogranin in human brain tissue. A cluster of peaks representing full-length Ng with different sets PTMs (**a**). Amino acid sequence of Ng1–78 with acetyl, disulfide bridge and GSH with the positions of PTMs and b- and y-ions identified from a single MS/MS acquisition indicated (**b**). Full-length Ng with different PTM arrangements had different retention time during high-resolution LC–MS/MS analysis (**c**). Expansion of the *m/z* range around *m/z* 7500 in a MALDI mass spectrum from human brain tissue after heat-treatment without reduction with DTT showed a cluster of peaks representing Ng1–78. The rightmost peak represents Ng1–78 + acetyl + GSH + disulfide bridge (**d**). A similar mass spectrum from human brain tissue after heat-treatment and reduction with DTT showed another cluster of peaks representing full-length Ng. Here the peak representing Ng1–78 + acetyl + GSH + disulfide bridge was greatly reduced (**e**)

Brain Ng was characterized using HI–MS and we found that Ng is present as a variety of short endogenous peptides, all located in the C-terminal part of the protein. In total, 15 peptides were repeatedly detected using MALDI TOF/TOF (Fig. 1) and an additional 24 peptides were identified by high-resolution mass spectrometry (Fig. 2 and Online Resource 3). By using a combination of HI–MS and high-resolution MS/MS we found that several post-translational modifications (PTMs) were present on full-length Ng. We were able to identify Ng1–78 with acetylation and a disulfide bridge, Ng1–78 with acetylation, disulfide bridge and cysteinyl, and Ng1–78 with acetylation, disulfide bridge and a glutathione (GSH) (Fig. 3a, c). In addition, all variants could also include one or two oxidations. A detailed data analysis showed that the disulfide bridge was located either between cysteine (C) 3 and C4 or between C4 and C9. GSH and cysteinyl may then be attached on either C9 or C3 depending on the location of the disulfide bridge. The acetylation was always attached to the protein N-terminal methionine M1 (Fig. 3b and Online Resource 4) and the oxidations to M1 or M41. Full-length Ng with the different sets of PTMs also had different retention time profiles (Fig. 3c). When reducing Ng with DTT the peaks representing Ng1–78 containing GSH or cysteinyl were greatly reduced (Fig. 3d, e). These results were confirmed with LC–MS/MS.

### The peptide-to-full-length neurogranin ratios are decreased in superior parietal gyrus of sporadic Alzheimer’s disease

**Fig. 4:**
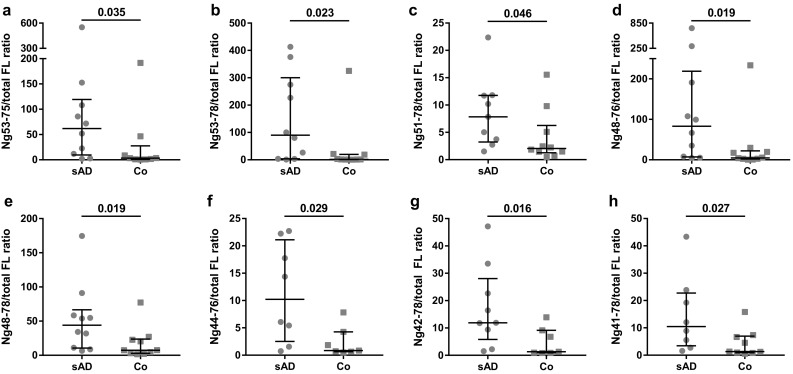
Scatterplots displaying the result from hybrid-immunoaffinity mass spectrometry in study 1. Scatterplots displaying the peptide-to-total full-length Ng ratio **×**1000 for Ng53–75 (**a**), Ng53–78 (**b**), Ng51–78 (**c**), Ng48–76 (**d**), Ng48–78 (**e**), Ng44–76 (**f**), Ng42–78 (**g**) and Ng41–78 (**h**). The data presented are median and interquartile ranges. Differences between groups were assessed using Mann–Whitney *U* test

In study 1, full-length Ng as well as 14 endogenous peptides, which were detected in all samples, was quantified in the superior parietal gyrus of individuals with sAD and healthy age-matched controls. After analyzing the ratio between each of the peptides and the sum of Ng1–78 with the three sets of PTMs, hereby referred to as total full-length Ng, we found that 8 peptide-to-total full-length Ng ratios were significantly increased in sAD compared to controls (*p* < 0.05 for all ratios) (Fig. 4 and Online Resource 5), thus indicating increased concentrations of these peptides compared to total full-length Ng.

### Neurogranin peptides are increased and full-length neurogranin is decreased in sporadic and familial Alzheimer’s disease

**Fig. 5:**
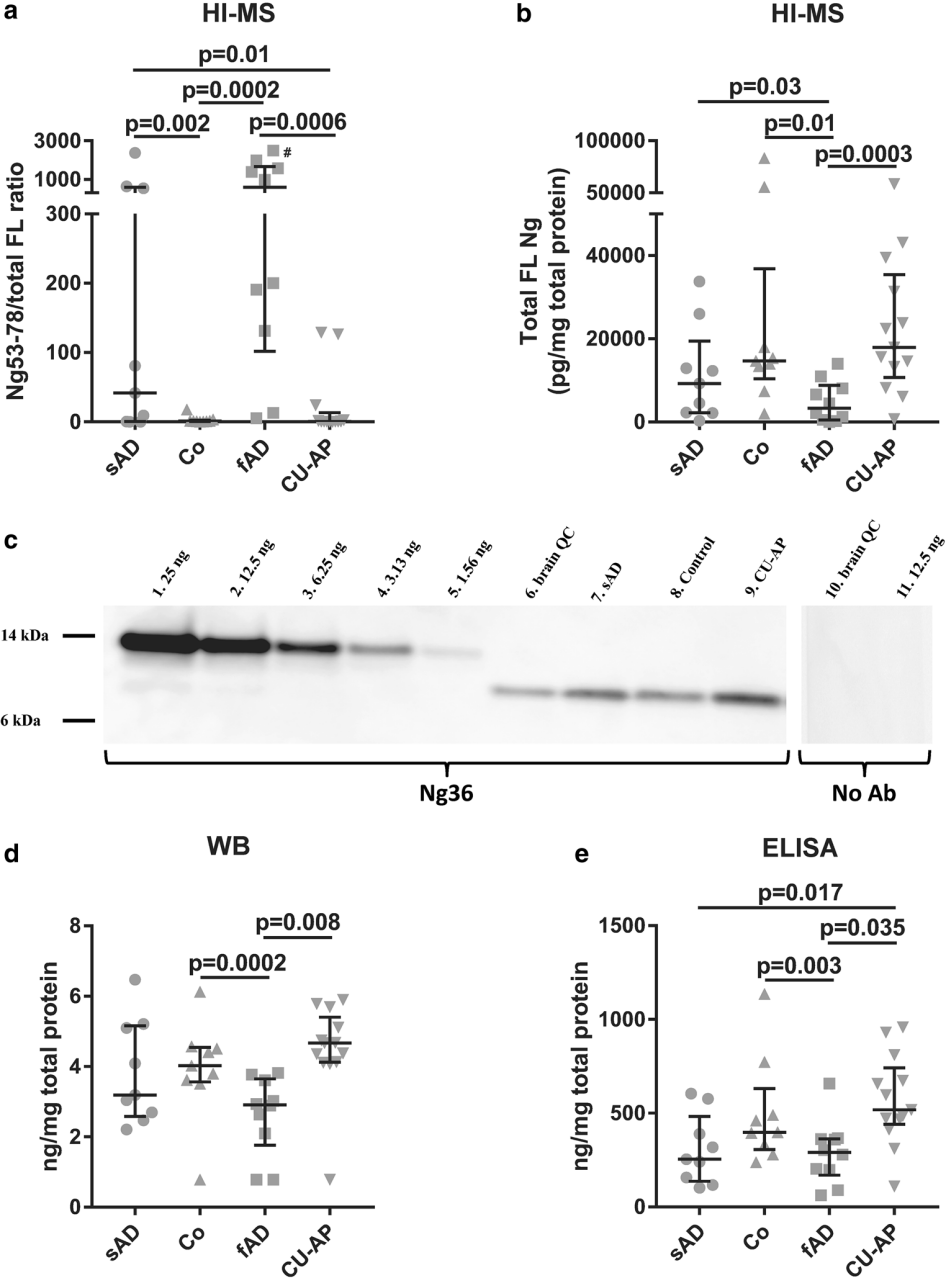
Scatterplots and western blot analysis of study 2. Scatterplot displaying the peptide-to-total full-length Ng ratio **×**1000 for Ng53–78. ^#^Sample ratio is > 150,000 (**a**). Scatterplot displaying total full-length Ng concentration measured by HI–MS (**b**). WB using the monoclonal antibody Ng36 (left blot) or no primary antibody (right blot). Briefly, samples were relatively quantified in WB by including a standard curve of recombinant Ng-MYC-DDK protein on each gel. Lanes 1–5: standard curve of Ng–Myc–DKK fusion protein ranging from 25 to 1.56 ng. Lane 6: quality control (QC) brain tissue sample. Lanes 7–9: patient samples from study 2. Lane 10: QC tissue sample. Lane 11: Ng–Myc–DKK fusion protein 12.5 ng. Lanes 10 and 11 were used as negative controls (**c**). Scatterplot displaying results from WB analysis. The *y*-axis displays the Ng concentration in ng/mg total protein in each sample (**d**). Scatterplot displaying ELISA results. The *y*-axis displays the Ng concentration in ng/mg total protein in each sample (**e**). The data presented are median and interquartile ranges. Comparisons between groups were performed using Kruskal–Wallis test with data adjusted for post-mortem delay, followed by pairwise Mann–Whitney *U* tests if significant

To validate these findings, we first used HI–MS in a second study with temporal cortex from individuals with sAD, fAD, CU-AP and controls. In total ten endogenous peptides, of which all except one were the same as in study 1, were quantified in a majority of the samples in addition to full-length Ng. There were significant differences across groups in peptide-to-total full-length Ng ratios for nine of the ten peptides detected in this study. One of the strongest differences was found in peptide-to-total full-length Ng ratio of Ng53–78 (*p *= 0.0008). For this peptide (Fig. 5a), a post hoc test revealed that peptide-to-total full-length Ng ratio was significantly reduced in sAD and fAD compared to controls (*p *= 0.004 for sAD; *p *= 0.0005 for fAD), and compared to CU-AP (*p *= 0.007 for sAD; *p *= 0.002 for fAD). Results for the other peptides are summarized in Online Resource 6 and 7.

Furthermore, there was a significant difference in total full-length Ng concentrations measured by HI–MS across groups (*p *= 0.0008). Post hoc analysis showed that total full-length Ng levels were significantly reduced in individuals with fAD compared to sAD (*p *= 0.03), CU-AP (*p *= 0.0003), and controls (*p *= 0.01) (Fig. 5b). There was also a trend, although not statistically significant, towards reduced total full-length Ng in sAD compared to controls and CU-AP.

In addition to HI–MS, the second cohort was also analyzed by WB (Fig. 5c) and ELISA. To ensure that the monoclonal antibody Ng36, which was used both in the ELISA and WB, was specific for Ng it was first characterized by HI–MS. Human brain tissue was analyzed by HI–MS and we were able to confirm that Ng36 is specific for Ng and detects the same peptides and full-length Ng as Ng2 and Ng3 (Online Resource 8).

For WB, there were significant differences across groups (*p *= 0.003), with again significantly lower concentrations in fAD compared to CU-AP (*p *= 0.008), and controls (*p *= 0.0002) (Fig. 5d). ELISA analysis also showed significant differences across groups (*p *= 0.008), with significantly lower concentrations in fAD compared to CU-AP (*p *= 0.035), and controls (*p *= 0.003), and significantly lower concentrations in sAD compared to CU-AP (*p *= 0.017) (Fig. 5e).

### Neurogranin levels differ between degrees of neuropathological changes

After grouping all individuals from study 2, independent of diagnosis, by Braak stages 0–I (no or little tau pathology), II–IV (moderate tau pathology), and V–VI (high-level tau pathology), we found a significant group difference in total full-length Ng, Ng measured by WB, Ng measured by ELISA, and peptide-to-total full-length Ng ratios of all peptides (all *p *< 0.02). Post hoc tests revealed that Ng levels increased with increasing Braak pathology for each of the tested measurements, results are summarized in Online Resource 9. After grouping individuals independent of diagnosis by Thal stages 0–1, 2–3, and 4–5, we found a significance group difference only in the peptide-to-total full-length Ng ratio of Ng53-78 (*p *= 0.03). A post hoc test showed that Ng values in Thal stages 0–1 were significantly lower than in stages 2–3 (*p *= 0.003) and nearly significantly lower than in stages 4–5 (*p* = 0.052). Results are summarized in Online Resource 9. Finally, after grouping individuals independent of diagnosis by CERAD score 0, A, B, and C, we found a significant group difference in total full-length Ng, Ng measured by WB, Ng measured by ELISA, and peptide-to-total full-length Ng ratios of all peptides (all *p *< 0.03). Again, post hoc tests showed increasing peptide-to-total full-length Ng ratios with increasing CERAD scoring. Results are summarized in Online Resource 9.

## Discussion

In 2015 we used HI–MS to show that several endogenous Ng peptides are present in both CSF and brain tissue and Ng48–76 was increased in sAD CSF [32] Therefore we aimed to investigate Ng in brain tissue in more detail by combining HI–MS, ELISA and WB analysis to find an explanation to why Ng seems to be decreased in sAD brains, as shown previously [15, 49], and increased in CSF from sAD patients. In the current study, we have performed an extensive characterization of Ng in post-mortem human brain tissue and quantified and compared the expression pattern of brain Ng in relation to AD pathology in sAD, fAD, and CU-AP to controls.

HI–MS combined with high-resolution MS/MS showed that full-length Ng is post-translationally modified by the addition of an N-terminal acetyl group, disulfide bridge (at C3–C4 or C4–C9), cysteinyl (at C3 or C9), and GSH (at C3 or C9), but does not exclude the possibility of other combinations of the reported variants, or different PTMs being present on full-length or nearly full-length Ng. Support of the reported PTMs include MS/MS data of the individual protein species and that the protein containing the different PTM combinations had different retention times. It has previously been shown that reducing samples with DTT prevents GSH adducts from forming [2]. The fact that the peak representing Ng1–78 with disulfide bridge and GSH was greatly diminished when reducing the sample with DTT add additional proof that GSH is present on Ng at a cysteine. Our results show that a substantial portion of full-length Ng has GSH as a PTM. GSH, which is the most abundant antioxidant in the brain, plays a significant role in counteracting oxidative stress by reacting with free radicals [43]. Proteins that are sensitive to redox might be protected from OS by glutathionylation and several proteins in AD brain, including p53 which can initiate apoptosis, have been identified as having a GSH PTM [19]. Loss of synapses is considered the mechanism that precedes neuronal loss and correlates best with cognitive impairment in AD [18, 51, 54, 57]. Both in vivo and in vitro studies suggest a direct relationship between synaptic dysfunction and oxidative stress in AD [14, 29, 35, 48], thus indicating that a GSH PTM on intact Ng might be a means of protection against oxidative stress to preserve protein function.

We were able to quantify several Ng peptides in both studies, although some of them differed between the two materials. This was most likely because the tissues used in the studies were from different regions. Using HI–MS we were able to quantify several peptides as well as full-length Ng with PTMs separately. In WB, the peptides were probably too small and exited the gel meaning that this method mainly quantifies full-length, or nearly full-length, Ng. In contrast, the ELISA most likely detects a mixture of both full-length Ng and many different peptides.

AD pathology (plaques and tangles) are common in older non-demented individuals, with an estimated 30–40% of cognitively intact elderly classed as positive upon autopsy [7, 31, 47]. Recently, new guidelines for diagnosis of AD based on biomarkers reflecting the key pathologies were published by the NIA and the AA to update and unify the 2011 NIA/AA guidelines according to the current understanding of the disease [40]. In the new 2018 NIA/AA guidelines for researchers, a diagnosis of AD is defined by its underlying pathologic processes that can be documented by post-mortem examination or followed in vivo using imaging or fluid biomarkers and not by symptoms or signs, such as cognitive decline, which are rather clinical consequences of the resulting neurodegeneration [27]. According to these criteria, the CU-AP would be classified as preclinical Alzheimer’s pathologic change in the Alzheimer’s continuum. In the present study, the sAD and CU-AP groups were different from each other with sAD having significantly higher concentrations for many of the measured Ng peptides compared to full-length Ng than CU-AP. In addition, the sAD and fAD subjects appear to be subjected to a similar processing of full-length Ng into peptides, whereas for controls and CU-AP the degradation is not so evident. Most importantly, the CU-AP individuals do not have any signs of lowered cognitive function despite mild to moderate neuropathological changes. Thus, grouping the AD and CU-AP subjects together would not be appropriate since CU-AP do not demonstrate an increase of Ng peptides and a decrease of full-length Ng, which might reflect degenerating synapses. In addition, differences between CU-AP and sAD have been described previously. Post-mortem brain tissue from half of the CU-AP individuals included in study 2 were previously analyzed and shown to contain less N-terminally truncated and pyroglutamate-modified Aβ peptides compared to sAD cases [44]. This would suggest that these individuals indeed are different from patients with sAD, and most likely also fAD, even though they share neuropathological changes in the brain.

Even though most CU-AP individuals have tau and amyloid pathology above what can be expected for their age they somehow still managed to remain cognitively intact. There is a possibility that these subjects were able to compensate for the synaptic damage and loss induced by these pathologies, thus keeping their full-length Ng levels in the same range as controls. Mouse models have shown that upregulation of Ng not only improves LTP but also enhances cognitive performance [65], hence suggesting that an increase in Ng might be beneficial for cognition in humans as well. As there was a trend towards increased full-length Ng in CU-AP compared to controls as measured by WB, it is possible that the CU-AP individuals are able to respond to the pathological changes and counteract or compensate for the cognitive decline these would otherwise cause, perhaps by increasing the numbers of synapses or simply producing more full-length Ng thus providing them with the means of a cognitive reserve.

As peptide-to-total full-length Ng ratios were increased in sAD compared to controls in both studies, and in the second study also for sAD compared to CU-AP and in fAD compared to both controls and CU-AP, there seems to be a shift from intact protein to endogenous peptides in both forms of AD. WB mainly detects full-length, or nearly full-length, Ng since the peptides appears to be too small and most likely exit the gel. Consequently, WB results also support the hypothesis of decreased full-length Ng as fAD had significantly lower concentrations compared to CU-AP as well as a trend towards lower levels compared to sAD. Similarly, the sAD group also had an apparent trend of lower concentrations compared to both controls and CU-AP in both WB and ELISA. In contrast to WB, the ELISA most likely detects a mixture of both full-length Ng and many different peptides as both Ng2 and Ng36 are able to bind Ng in the form of both peptides and full-length protein. This might in turn explain why the group separation was less evident compared to WB. However, HI–MS analysis showed that even though peptide concentrations are increased in both sAD and fAD there is still very high levels of full-length Ng left, which is most likely why the ELISA results still are more similar to WB than HI–MS. Taken together, this shows that full-length Ng most likely is degraded into at least some of the peptides measured by HI–MS. The results from the ELISA are very similar to WB with significantly lower concentrations in fAD compared to CU-AP and, although not reaching statistical significance, a trend towards lower concentrations in AD compared to both controls and CU-AP and as well as when comparing fAD with controls. Thus, the three methods indicate the same conclusion; that full-length Ng is decreased in both sAD and fAD.

Since we saw clear differences in the peptide-to-total full-length Ng ratios between the two AD groups compared to controls and CU-AP for many of the detected peptides, it might be of potential interest to develop an assay capable of measuring these two pools of Ng in the future. Currently, full-length and peptide Ng are measured by HI–MS, which is both quite time-consuming and inefficient compared to an immunoassay. The ELISA presented here most likely quantifies both full-length and peptide Ng, and the separation between the groups is not as good compared to peptide-to-total full-length Ng ratio. In theory, it might be possible to develop two assays that quantify peptide and full-length Ng, respectively. Using a similar approach, the Aβ42/40 ratio has successfully been shown to increase diagnostic accuracy [22, 34, 56] and have diagnostic value in clinical settings [21], as well as circumventing the issue of high- and low-producers [62].

It is well established that neuronal and synaptic loss are early and central events in AD pathology [18, 37, 52] and that synaptic density is reduced by more than 30% even in the earliest stages of AD [16]. In fact, both cognitive decline and disease progression can be monitored by measuring synaptic loss as it appears to be more closely correlated with cognitive deficits than plaque and tangle load [37, 52, 58]. In rodents, Ng mRNA and protein concentrations in the hippocampus decrease with age and are related to CNS dysfunction [41] and knockdown models display both impaired cognition and reduced LTP [23]. In humans, it has been shown that high CSF Ng was positively associated with increased rate of hippocampal atrophy [46] as well as other parts of the brain [59]. Taken together, it seems probable that the proteolytic processing of full-length Ng into peptides during neurodegeneration, visualized here as increased peptide-to-total full-length Ng concentrations in sAD and fAD, may be triggered by synaptic degeneration and neuronal loss. According to the literature, at present day Ng does not seem to be a confirmed substrate for any enzymes. However, recently we showed that that calpain-1 and prolyl endopeptidase are capable of cleaving full-length Ng within the IQ domain and near the C-terminal end, respectively in vitro resulting in several of the endogenous peptides we have found in brain tissue and CSF [5]. Notably, several of the endogenous peptides identified from brain tissue start just after the C-terminal end of the IQ motif. Since the IQ motif is needed for calmodulin-binding [3, 50], the cleavage would most likely inhibit Ng binding to calmodulin, as it does for GAP-43 [64], which in turn would affect LTP and therefore formation of long-term memory excitation [4, 20, 25, 63]. Future studies on the role of this enzymatic activity would be of interest to further understand the link between neurodegeneration and enzymatic cleavage of Ng into the observed peptides as they are not all accounted for by cleavage of calpain-1 and prolyl endopeptidase alone.

We previously showed a strong association between CSF Ng and degree of AD neuropathology independent of diagnosis in a study containing several neurodegenerative diseases [45]. In the present study, we confirmed the CSF study as brain Ng levels were increasing with both Braak stage, CERAD score and to some extent Thal phase, meaning that Ng is associated with both Aβ and tau pathology. However, CERAD scores showed a much stronger association with Ng levels compared to Thal phases. This may be due to the fact that Thal phases are a measure of how far the Aβ pathology has spread around the brain, regardless of how much Aβ is present, whereas the CERAD score is a measure of the quantity of Aβ plaques found in the cortical areas. For example, to reach a Thal phase 1, Aβ plaques must be present in any cortical region, but this gives no information at all about the actual number of plaques present. In conclusion, our data shows that Ng is much more closely associated with plaque load compared to the spreading of Aβ pathology. This is in line with previous findings that CSF Ng is positively correlated with CERAD scores in autopsy-confirmed cases of AD [45]. Evaluating the levels of Ng in CSF from patients with primary tauopathies, without amyloid plaque pathology, might give us further insight into the association with tau as previous studies on CSF Ng have shown strong correlations with CSF tau [17, 24, 30, 33, 38, 61].

The major limitations of this paper are the small sample sizes in both studies and significant age differences between the groups in study 2. The sample sizes are limited since high-quality post-mortem tissue meeting the neuropathological demands of the groups included in these studies is very restricted. Regarding age differences in study 2, fAD was significantly younger than both controls and CU-AP, and sAD was younger than CU-AP. However, as sAD and controls were age-matched in both studies, and there was no significant difference in age between controls and CU-AP, it is unlikely that the reported differences in Ng concentrations are due to the age differences. Since this study was performed on post-mortem tissue, it was difficult to ensure age matching between AD and control groups of the two separate studies as well as all four groups included in the second study, especially considering the fAD group will be younger than the sAD group. To compensate for any impact the age differences may have on the results, all data was adjusted for age. Post-mortem delay was significantly shorter in fAD compared to CU-AP therefore statistical analysis were adjusted for this.

We have performed an extensive characterization of the postsynaptic protein Ng in human brain tissue. Using HI–MS it was shown that Ng is present as a variety of endogenous peptides as well as post-translationally modified full-length protein. The identified PTMs were acetyl, disulfide bridge, cysteinyl, and GSH modifications, as well as oxidation. The most important discovery was that sAD and fAD had increased concentrations of endogenous peptides as well as a decrease in full-length Ng indicating a shift from intact protein towards peptides. This shift was not present in healthy controls or CU-AP individuals, of which the latter have both plaque and tangle pathology but no cognitive impairment, hence indicating that Ng is a biomarker for AD-related synaptic degeneration that leads to cognitive decline. This was also supported by the finding that Ng levels were strongly associated with degree of neuropathological changes, as measured by Braak stage, Thal phases, and CERAD scores, independent of diagnosis.

## Electronic supplementary material

Below is the link to the electronic supplementary material.

Detailed demographics of all subjects included in study 1 and 2 (XLSX 19 kb)

Supplementary Materials and methods (DOCX 22 kb)

Summary of all neurogranin peptides identified by using hybrid–immunoaffinity mass spectrometry. Deviation (ppm) = relative mass deviation = (measured mass − theoretical mass)/theoretical mass (XLSX 14 kb)

MS/MS spectrum of post-translational modifications on full-length neurogranin. High-resolution MS/MS spectrum of Ng 1–78 with disulfide bridge between C4 and C9 (a), disulfide bridge between C3 and C4 (b), disulfide bridge between C4 and C9 and cysteinyl on C3 (c), disulfide bridge between C3 and C4 and cysteinyl on C9 (d), disulfide bridge between C4 and C9 and glutathione (GSH) on C3 (e) and disulfide bridge between C3 and C4 and GSH on C9 (f) (TIFF 6013 kb)

Summary of results and *p* values from study 1. *p* values for the peptide-to-total full-length Ng ratios in the first study. Comparisons between groups were performed using the non-parametric Mann–Whitney *U* test. Significant *p* values are marked in bold font. *These peptides have the exact same mass and can therefore not be distinguished during sample analysis (XLSX 12 kb)

Scatterplots displaying the result from hybrid–immunoaffinity mass spectrometry in study 2. Scatterplots displaying the peptide-to-total full-length Ng ratio **×**1000 for Ng53–75 *Sample ratio is > 60,000 (a), Ng53–76#Sample ratio is > 400,000 (b), Ng52–78 ^#^Sample ratio is > 500 (c), Ng48–75 + 49–76 ^#^Sample ratio is > 45,000 (d), Ng48–76 ^#^Sample ratio is > 200,000 (e), Ng48–78 ^#^Sample ratio is > 15,000 (f), Ng46–78 ^#^Sample ratio is > 8000 (g), Ng44–78 ^#^Sample ratio is > 8000 (h). The data presented are median and interquartile ranges. Differences between groups were assessed using Mann–Whitney *U* test. (TIFF 871 kb)

Summary of results and *p* values from study 2. *p* values for the peptide-to-total full-length Ng ratios in the second study. Comparisons between groups were performed using Kruskal–Wallis test with data adjusted for post-mortem delay, followed by pairwise Mann–Whitney *U* tests if significant. *These peptides have the exact same mass and can therefore not be distinguished during sample analysis. §These peptides have the exact same mass and can therefore not be distinguished during sample analysis (XLSX 13 kb)

Hybrid immunoaffinity–mass spectrometric characterization of Ng36 in brain tissue. Several short endogenous C-terminal Ng peptides were repeatedly detected in human brain tissue using Ng36 in HI–MS combined MALDI TOF/TOF. Ng43–75* represents internal standard fully labeled with ^13^C (a). A cluster of peaks representing full-length and post-translationally modified Ng (b) (TIFF 1039 kb)

Summary of *p* values for association between neurogranin and neuropathological scores. *p* values for the correlation of Ng between different levels of neuropathological changes. Comparisons between groups were performed using Kruskal–Wallis test with data adjusted for post-mortem delay, followed by pairwise Mann–Whitney *U* tests if significant. Significant *p* values are marked in bold font. *These peptides have the exact same mass and can therefore not be distinguished during sample analysis (XLSX 15 kb)
