# Supplementary material for: The intact postsynaptic protein neurogranin is reduced in brain tissue from patients with familial and sporadic Alzheimer’s disease
Source: Acta Neuropathol. 2018 Sep 22;137(1):89–102. doi: 10.1007/s00401-018-1910-3 (PMC6338696; doi:10.1007/s00401-018-1910-3)
Supplement: Supplementary file 2 — Supplementary Materials and methods (DOCX 22 kb) [file 401_2018_1910_MOESM2_ESM.docx]

Supplementary Material and Methods

Sample reconstitution for LC-MS/MS

For analysis of immunopurified brain extracts, samples were reconstituted in 7 µL 8% FA/8% ANC in water (v/v/v) vortexed briefly and but on shake 30-60 min. All of the sample solution was then transferred to LC-vials for analysis by nanoflow LC-MS/MS.

LC-MS/MS

Analysis of immunopurified brain extracts was performed with nanoflow LC coupled to electrospray ionization (ESI) orbitrap MS and MS/MS. This was done with a Dionex Ultimate 3000 system (solvent rack SRD-3400, pump NCS-3500RS, autosampler WPS-3000 TPLRS) coupled to a Q Exactive equipped with a Nanospray Flex ion source. Six µL sample was injected onto a reversed-phase Acclaim PepMap C18 (length 20 mm, i.d. 75 µm, particle size 3 µm, pore size 100 Å) trap column used for online desalting and sample clean-up, followed by a reversed-phase Acclaim PepMap RSLC C18 (length 150 mm, i.d. 75 µm, particle size 2 µm, pore size 100 Å, both Thermo Fisher Scientific) for separation. The separation was performed at a flow rate of 300 nL/min by applying a linear gradient of 0-40% B for 50 min. Mobile phase A was 0.1% FA in water (v/v) and mobile phase B was 0.1% FA/84% ACN in water (v/v/v). Mass spectra were acquired in positive ion mode with a voltage setting of +1.7 kV and a resolution setting of 70,000 target values were 1×10^6^ both for MS and MS/MS acquisitions. Typical acquisitions were performed with an *m/z* range of 400–1800 with 1 microscan/acquisition. Precursor isolation width was 3 *m/z* units and peptide match was deselected. Singly and doubly charged ions and ions with unassigned charge were deselected for fragmentation. The instrument was operated in data dependent mode so that each MS acquisition was followed by 5 fragment ion acquisitions of the 5 most intense ions, which were fragmented by so-called higher energy collision induced dissociation (HCD) at a normalized collision energy (NCE) of 25. Selected *m/z* were excluded for 5 s until eligible again.

Data analysis

Mass spectra containing larger, multiply charged species, such as full-length Ng, require redetermination of the precursor ion monoisotopic *m/z* and charge-deconvolution of the peaks. Database searches were performed with two different softwares. This was accoplished by Mascot Daemon v2.6.0 combined with Mascot Distiller v.2.6.3 (both Matrix Science, London, UK) as well as with Peaks Studio v8.5. For Proteome Discoverer and Mascot Deamon/Distiller searches were submitted to the in-house Mascot database server (v2.6.1) while for Peaks Studio the search feature was built-in. See below for parameter settings for the different softwares. The data output from both softwares was also subjected to extensive manual evaluation.

LC and MS software

The aquisition software used was Thermo Foundation 3.1.64.11 with Xcalibur 3.1.66.10 and instrument configuration Q Exactive - Orbitrap MS 2.8 SP1 build 2806. Both LC systems were connected through Thermo Scientific SII for Xcalibur 1.3.0.73 with Chromeleon 7.2.4.8179 for LC control.

Data processing and database search settings

Mascot Daemon search parameters – search using Mascot Distiller for spectrum processing

All searches [Database = custom made Ng, Uniprot_SwissProt; Fixed modifications = none; Variable modifications = Oxidation (M), Acetyl (N-term), Cysteinyl (C), Dehydro (C), Glutathione (C); Decoy = not enabled; Enzyme = none; Max. missed cleavages = not relevant; Monoisotopic = selected; Peptide charge = 1+; Peptide tol. = 20 ppm; #13C = 0].

MS/MS [MS/MS ion search = enabled; Error tolerant search = not enabled; Data format = Mascot generic; MS/MS tolerance = 50 mmu; Quantitation = none; instrument type = CID fragment type spectra – all singly charged *b*- and *y*-ions].

Data import filter = Mascot Distiller [Data File Format = ThermoXcalibur; Default for unknown scan type = Profile/continuum; Data import filter options: Mascot Distiller Processing Options = see below; Multi-Sample Files = Separate search for each sample; Peak List Format = MGF; Intensity values = Area; Scan Range (multi-scan files) = sample dependent; Distiller Project File Save = enabled; Output PMF Masses as = MH+; Output MS/MS Fragments as = MH+].

Mascot Distiller Processing Options

MS Processing: Un-centroiding [Peak half width = 0.025; Data points per Da = 100; Data points per Da = 100; Always uncentroid = enabled]; Re-gridding [Data points per Da = 100]; Multi-Format Spectrum [Preferred type = Profile]; Peaks [Minimum number = 1; Maximum charge = 15]; Aggregation [Scan group aggregation method = Sum]. MS/MS Processing: Un-centroiding [Peak half width = 0.025; Data points per Da = 100; Data points per Da = 100; Always uncentroid = enabled]; Re-gridding [Data points per Da = 100]; Multi-Format Spectrum [Preferred type = Profile]; Peaks [Minimum number = 8; Maximum charge = N/A; Use precursor charge as maximum = enabled]; Precursor Charge [1st choice = Try to re-determine charge from parent scan; 2nd choice = If available, take charge from file; 3rd choice = Use default charge(s); Default charge range = 2 to 15; Ignore singly charged precursors = not enabled]; Precursor Selection [Search within m/z tolerance of = 2.5 Da; Re-determine precursor m/z value when possible = enabled; Maximum number of precursor m/z values = 1]; Aggregation [Scan group aggregation method = Time Domain].

Time Domain: Group assignment [Minimum precursor mass (Mr) = 700; Maximum precursor mass (Mr) = 16 000; Precursor m/z tolerance = 0.01; Maximum intermediate time (secs) = 0; Maximum intermediate scan count = dimmed; Use intermediate scan count when possible = not enabled]; Group Filtering [Minimum number of scans = 1]. Group combination [Sum MSn scans into MS2 = enabled]. MS Peak Picking: Filtering [Correlation threshold (Rho) = 0.5; Minimum signal to noise (S/N) = 5; Minimum peak m/z = 50; Maximum peak m/z = 100 000]; Peak profile [Minimum peak width (Da) = 0.001; Expected peak width (Da) = 0.025; Maximum peak width (Da) = 0.5; Reject width outliers = not enabled]; General [Apply baseline correction = not enabled; Fit method = Isotope Distribution; Maximum peak iterations per scan = 500]; Single Peak Window [Pick single peaks in this range (e.g. reporter ions) = not enabled; other parameters dimmed]. MS/MS Peak Picking: General [Same as MS Peak Picking = enabled; other parameters dimmed].

PEAKS Studio 8.5 processing parameters

Create Project options: [Instrument type = Orbitrap (Orbi-Orbi); Fragment = HCD]. Data Refinement options: [Merge Scans = not enabled; Correct Precursor = enabled; Mass only (recommended) = enabled; Filter Scans = not enabled]. Identification options: [Error Tolerance: Precursor mass = 20 ppm using monoisotopic mass; Fragment ions = 0.05 Da; Enzyme = None; Selected Fixed PTM = none; Selected Variable PTM = Oxidation (M), Acetylation (Protein N-term), Cysteinylation, Glutathione disulfide, Half of a disulfide bridge; max variable PTM per peptide = 6; Database = custom made Ng database; Uniprot_SwissProt].
