# Supplementary figures and images for: The intact postsynaptic protein neurogranin is reduced in brain tissue from patients with familial and sporadic Alzheimer’s disease
Source: Acta Neuropathol. 2018 Sep 22;137(1):89–102. doi: 10.1007/s00401-018-1910-3 (PMC6338696; doi:10.1007/s00401-018-1910-3)

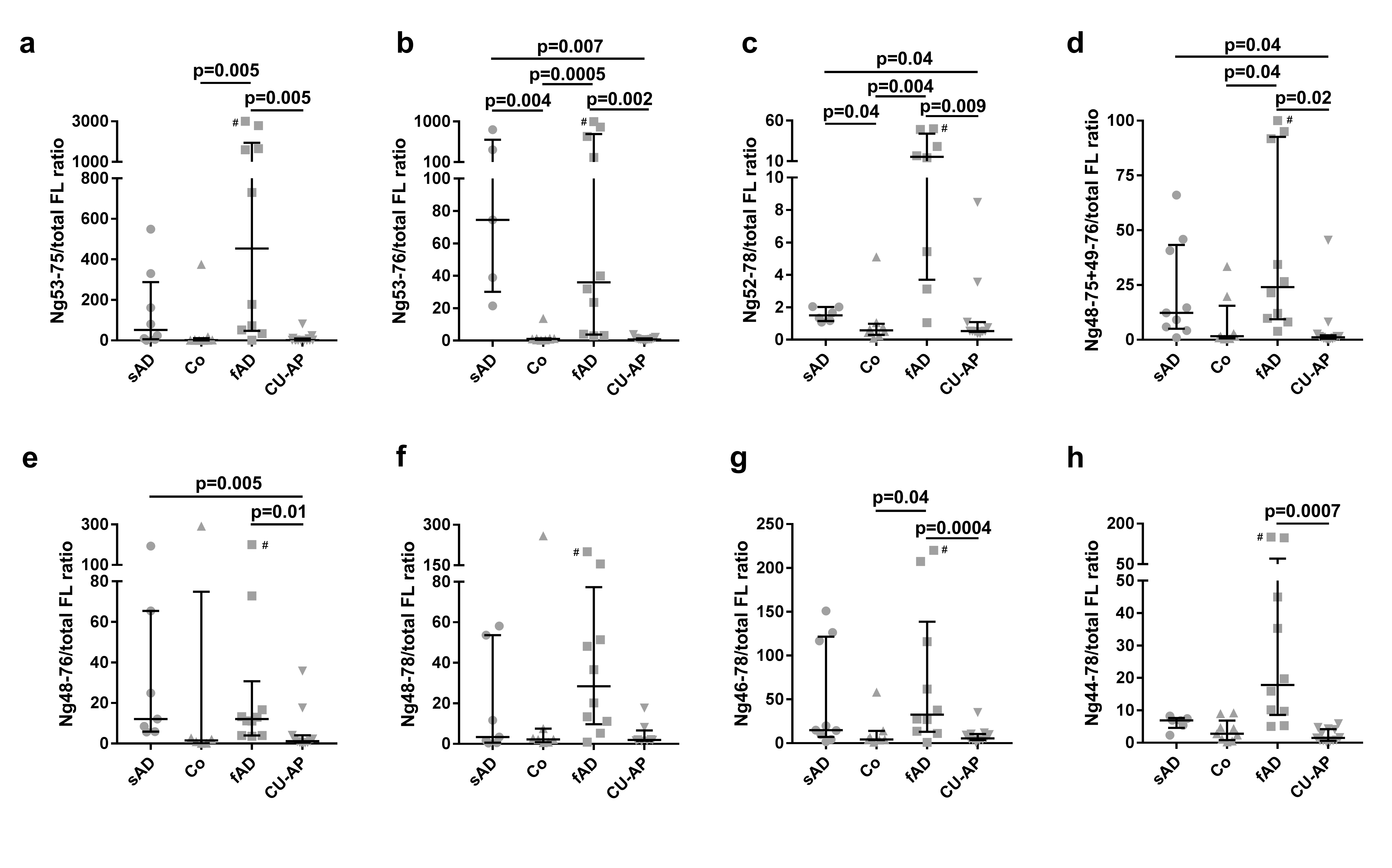

Supplement: Supplementary file 6 — Scatterplots displaying the result from hybrid–immunoaffinity mass spectrometry in study 2. Scatterplots displaying the peptide-to-total full-length Ng ratio ×1000 for Ng53–75 *Sample ratio is > 60,000 (a), Ng53–76#Sample ratio is > 400,000 (b), Ng52–78 #Sample ratio is > 500 (c), Ng48–75 + 49–76 #Sample ratio is > 45,000 (d), Ng48–76 #Sample ratio is > 200,000 (e), Ng48–78 #Sample ratio is > 15,000 (f), Ng46–78 #Sample ratio is > 8000 (g), Ng44–78 #Sample ratio is > 8000 (h). The data presented are median and interquartile ranges. Differences between groups were assessed using Mann–Whitney U test. (TIFF 871 kb) [file 401_2018_1910_MOESM6_ESM.tif]

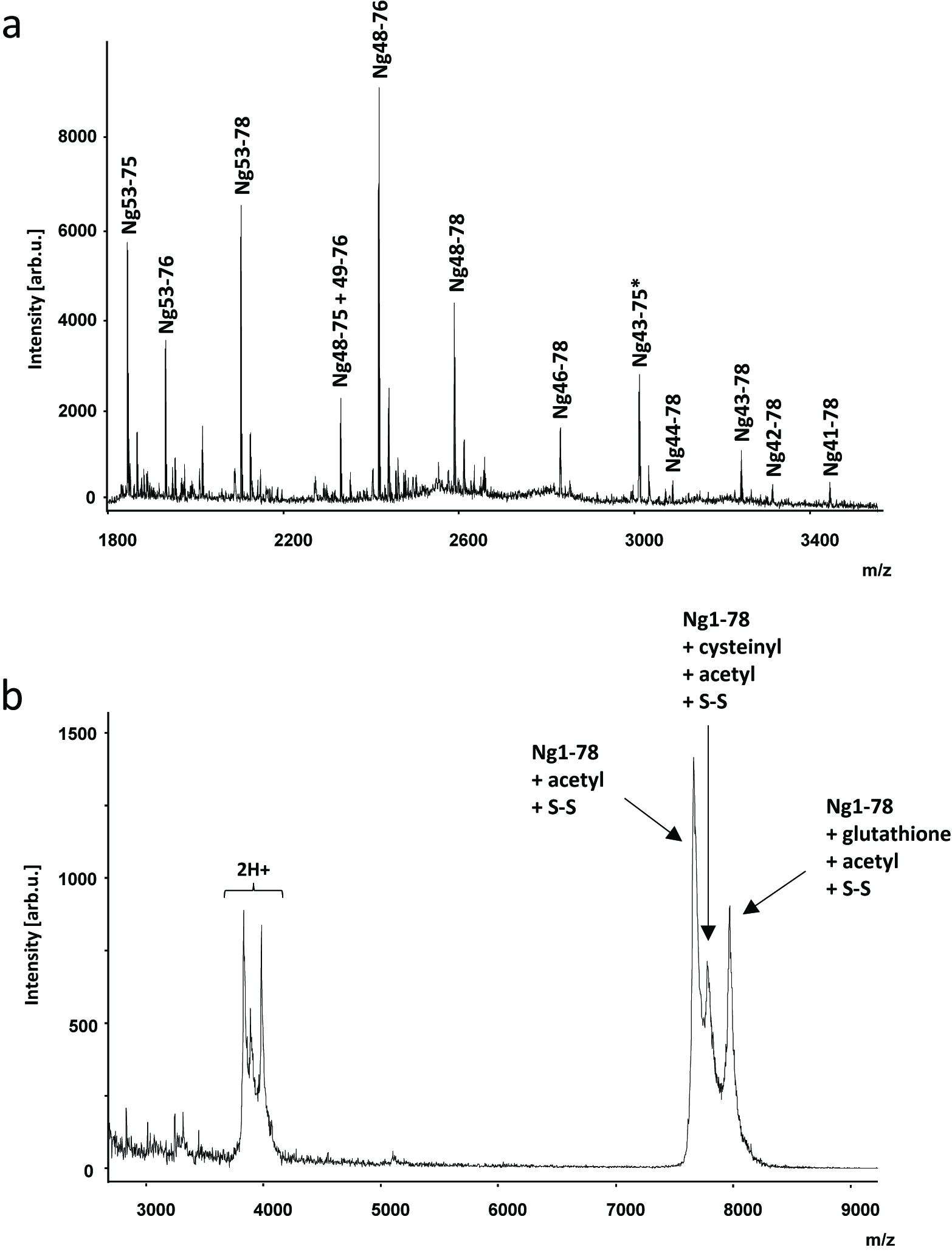

Supplement: Supplementary file 8 — Hybrid immunoaffinity–mass spectrometric characterization of Ng36 in brain tissue. Several short endogenous C-terminal Ng peptides were repeatedly detected in human brain tissue using Ng36 in HI–MS combined MALDI TOF/TOF. Ng43–75* represents internal standard fully labeled with 13C (a). A cluster of peaks representing full-length and post-translationally modified Ng (b) (TIFF 1039 kb) [file 401_2018_1910_MOESM8_ESM.tif]
